# Impact of biology knowledge on the conservation and management of large pelagic sharks

**DOI:** 10.1038/s41598-017-09427-3

**Published:** 2017-09-06

**Authors:** Hiroki Yokoi, Hirotaka Ijima, Seiji Ohshimo, Kotaro Yokawa

**Affiliations:** National Research Institute of Far Seas Fisheries, Japan Fisheries Research and Education Agency; 5-7-1 Orido Shimizu, Shizuoka city, Japan

## Abstract

Population growth rate, which depends on several biological parameters, is valuable information for the conservation and management of pelagic sharks, such as blue and shortfin mako sharks. However, reported biological parameters for estimating the population growth rates of these sharks differ by sex and display large variability. To estimate the appropriate population growth rate and clarify relationships between growth rate and relevant biological parameters, we developed a two-sex age-structured matrix population model and estimated the population growth rate using combinations of biological parameters. We addressed elasticity analysis and clarified the population growth rate sensitivity. For the blue shark, the estimated median population growth rate was 0.384 with a range of minimum and maximum values of 0.195–0.533, whereas those values of the shortfin mako shark were 0.102 and 0.007–0.318, respectively. The maturity age of male sharks had the largest impact for blue sharks, whereas that of female sharks had the largest impact for shortfin mako sharks. Hypotheses for the survival process of sharks also had a large impact on the population growth rate estimation. Both shark maturity age and survival rate were based on ageing validation data, indicating the importance of validating the quality of these data for the conservation and management of large pelagic sharks.

## Introduction

With the increasing exploitation of oceans globally, shark conservation and management have become crucial^1–4^. Sharks are vulnerable to extinction and recover slowly from stock exploitation^5, 6^. The population increase of some sharks is very slow due to their late sexual maturity, low fecundity, small litter size and slow growth rate^6, 7^. Particularly, the oceanic pelagic shark population has reduced globally, due to an increase in fishing pressure^8–12^. In the trophic chain, pelagic sharks are an apex species with structural and functional importance in the large pelagic oceanic ecosystem^13, 14^. Several studies have indicated that trophic cascades might be occurring in marine ecosystems because of the effects from changes in predator abundance^15–18^. Thus, conservation of pelagic asharks is important from an ecological perspective and to protect the biodiversity and ecosystem structure and function.

The blue shark (*Prionace glauca*) and shortfin mako shark (*Isurus oxyrinchus*) are common sharks in pelagic ecosystems. They are caught internationally by longline and gillnet fishing as bycatch in tuna fisheries and in targeted fisheries in the Exclusive Economic Zone (EEZ) and the high seas^19–21^. Blue sharks are a key species in the pelagic ecosystem because they are caught in the largest numbers among pelagic shark fisheries^4, 13^. The shortfin mako shark is considered Vulnerable (VU) species by the International Union for Conservation of Nature (IUCN)^22, 23^. To implement appropriate conservation and management of these sharks, tuna Regional Fisheries Management Organizations (RFMOs) have been conducting stock assessments of each region^24–26^. However, the estimated stock status of pelagic sharks, such as estimated biomass and maximum sustainable yield (MSY), include significant uncertainties^25^. A common reason for this uncertainty is inaccurate catch statistics because pelagic sharks are often discarded or caught by illegal fisheries^27–29^.

Population growth rates are used as relevant information for the conservation and management of natural resources^30–32^. In particular, the population growth rate is a crucial parameter for determining the vulnerability of population decline^33^ and is defined as the intrinsic rate of population increase using the eigenvalue matrix population model *r* = ln(*λ*), which is calculated based on life history parameters^34, 35^. Life history parameters are elements of matrix models and comprise biological parameters, such as natural mortality, longevity, fecundity, mating system and maturation. If the correlation between the population growth rate and these biological parameters is well understood, rational plans for conservation and management can be developed. Sensitivity and elasticity analyses are useful tools that affect absolute and proportional life history parameter changes related to population growth rate^36, 37^. Precise biological parameters lead to realistic population growth rates, which can be used for stock assessment and ecological risk assessment (ERA). In detail, population growth rate can be used for prior parameter of stock assessment models such as the Bayesian surplus production model (BSPM). In the ERA framework, population growth rate has been used in the productivity component of the productivity and susceptibility analysis (PSA), which is a useful methodology for the conservation of sharks when the stock status cannot be estimated^38, 39^. Thus, these methods represent a logical first step in the conservation and management of pelagic sharks.

**Figure 1:**
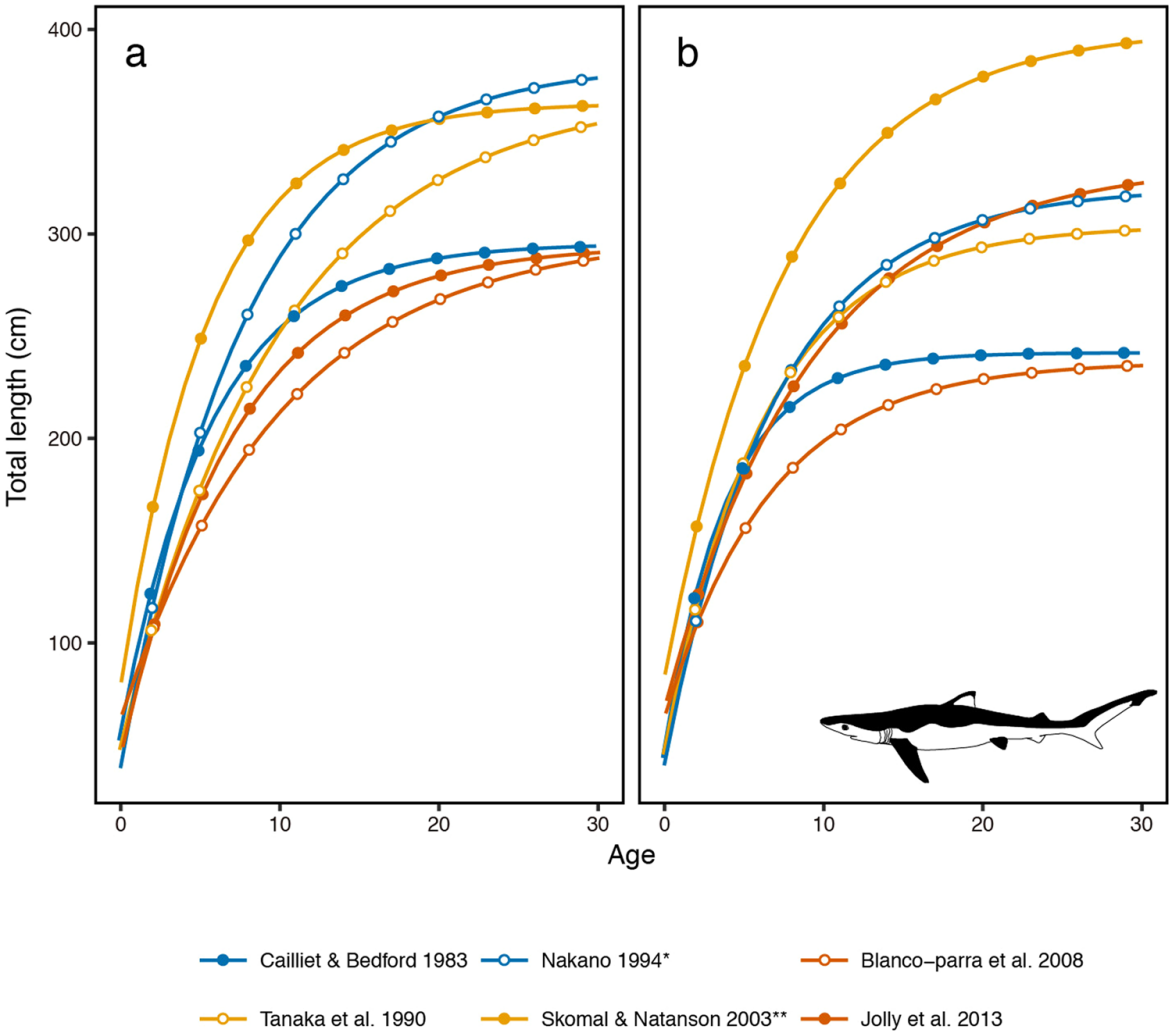
The estimated growth curve of the blue shark. (**a**) Growth of male sharks. (**b**) Growth of female sharks. *Precaudal lengths were converted to total lengths. **Fork lengths were converted to total lengths.

**Figure 2:**
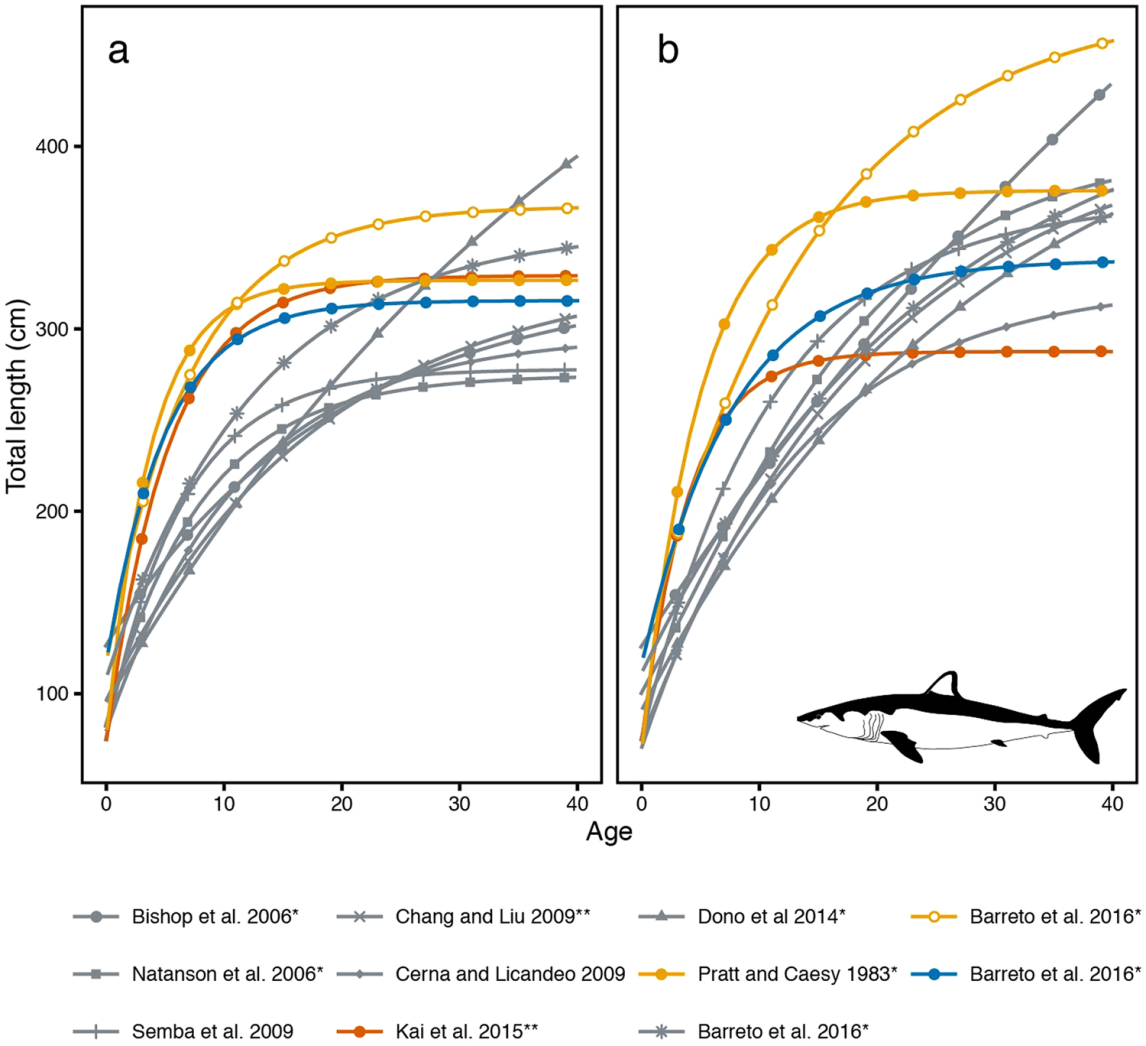
The estimated growth curve of the shortfin mako shark. (**a**) Growth of male sharks. (**b**) Growth of female sharks. *Fork lengths were converted to total lengths. **Precaudal lengths were converted to total lengths. Grey lines represent estimates from one band-pair measurement annually. Yellow lines represent estimates from two band-pair measurements annually. The blue line represents estimates from two band-pair measurements annually until five years old. The red line represents estimates using length composition data.

Estimating accurate population growth rates of blue and shortfin mako sharks has been difficult, as the biological parameters of both pelagic sharks are not well known, particularly for growth curves (Figs 1 and 2). In addition, for shortfin mako sharks, the biological parameters differ substantially by sex^40–45^. Several studies have estimated the population growth rate of blue and shortfin mako sharks using various methods^7, 25, 39, 46–50^. A PSA analysis was conducted by developing a simple age-structured Leslie matrix model considering the uncertainty of life history parameters^51^. This Leslie matrix model was applied only to females. To evaluate sex-dependent effects on the population growth rate, a stage- and sex-based matrix population model for the shortfin mako shark was developed and explored using a stochastic simulation with the uncertainty of life history parameters^49^. The effect of the mating system on the population growth rate of the shortfin mako shark was also evaluated using a two-sex stage-structured matrix population model with different mating systems^50^. However, they did not concretely assess the effects of differing biological parameters. To reflect the actual life cycle of pelagic sharks, a two-sex age-structured matrix population model is more appropriate, as these biological parameters differ by sex. Furthermore, it is necessary to clarify what kinds of biological parameters are critical when estimating the population growth rate. This information would clarify the most important biological study for the conservation of both pelagic sharks. Several stochastic analyses were exported to discuss the uncertainty of life history parameters for population growth rate that assumed annual change of biological parameters such as natural mortality^30, 51^. However, for some life history parameters, there are uncertainties associated with the quality of estimation (e.g., maturity at age) rather than the stochastic change such as the annual change in natural mortality. We considered that it is better to assess the quality of estimation to define the most critical biological parameter. Therefore, in this study, we established a two-sex age-structured matrix population model and focused on the effect quality of parameter estimation. Concretely, we calculated the population growth rates of blue and shortfin mako sharks using the two-sex age-structured matrix population model and available biological parameter combinations. We compared these estimated population growth rates with previous studies. Moreover, we summarised the population growth rate by each parameter to clarify the effect of the biological parameters. We also performed an elasticity analysis to observe the impact of life history parameters on the population growth rate of pelagic sharks.

## Results

### Population growth rate

The estimated median value of the long-term population growth rate of the blue shark was 0.384, ranging within 0.195–0.533 (Table 1). For comparison, the highest and lowest reported population growth rates are 0.599^46^ and 0.169^46^, respectively (Table 1). Thus, our results did not exceed the range of previously estimated population growth rates for the blue shark. For the shortfin mako shark, the estimated median value of the population growth rate was 0.102, ranging within 0.007–0.318 (Table 1). The highest population growth rate estimated in this study (0.318) is greater than that estimated in a previous study (0.170)^47^ (Table 1). Similarly, the lowest population growth rate estimated in this study also exceeds that reported in a previous study (0.010)^50^] (Table 1).

**Table 1:** Estimated population growth rates (*r*) of blue shark (*Prionace glauca*) and shortfin mako shark (*Isurus oxyrinchus*).

Species	Value	Covered area	Model/Method	Reference
Blue shark	median = 0.384 (0.195–0.533)^*a*^	Global	Two-sex age-structured matrix population model	**This study**
	0.28–0.41^*b*^	North Pacific	Bayesian surplus production model	25
	mean = *0.337 (0.250–0.428)^*c*^	North Atlantic	Age-structured matrix population model	39
	median = 0.286 (0.237–0.334)^*c*^	North Atlantic	Age-structured matrix population model	51
	mean = 0.30, sd = 0.045	North Pacific	Bayesian surplus production model	80
	0.169–0.599^*b*^	North Pacific	Euler-Lotka equation	46
	mean = *0.297 (0.214–0.373)^*d*^	North West Pacific	Euler-Lotka equation	47
	mean = *0.259 (0.198–0.317)^*d*^	North East Pacific	Euler-Lotka equation	47
Shortfin mako shark	median = 0.102 (0.007–0.318)^*a*^	Global	Two-sex age-structured matrix population model	**This study**
	mean = *0.132 (0.093–0.166)^*c*^	North Atlantic	Age-structured matrix population model	39
	0.014	Atlantic	Euler-Lotka equation	48
	0.073	Atlantic	Age-structured matrix population model	51
	0.058–0.059^*b*^	North Atlantic	Bayesian surplus production model	26
	0.058–0.062^*b*^	South Atlantic	Bayesian surplus production model	26
	*0.075, 0.050	North Pacific	Two-sex stage-structured matrix population model	49
	*0.010–0.079^*b*^	North Pacific	Two-sex stage-structured matrix population model	50
	mean = *0.030 (−0.047–0.101)^*d*^	North Pacific	Euler-Lotka equation	47
	mean = *0.114 (0.056–0.170)^*d*^	California	Euler-Lotka equation	47

^a^The range of limits denotes maximum and minimum population growth rates. ^b^Population growth rate estimated by several scenarios. ^c^The range of limits denotes the 2.5th and 97.5th percentiles. ^d^The range of limits indicates the 95 percent confidence interval. *Eigenvalue (*λ*) of the matrix population model was estimated, which we converted to population growth rate (*r*).

### Elasticity analysis

**Figure 3:**
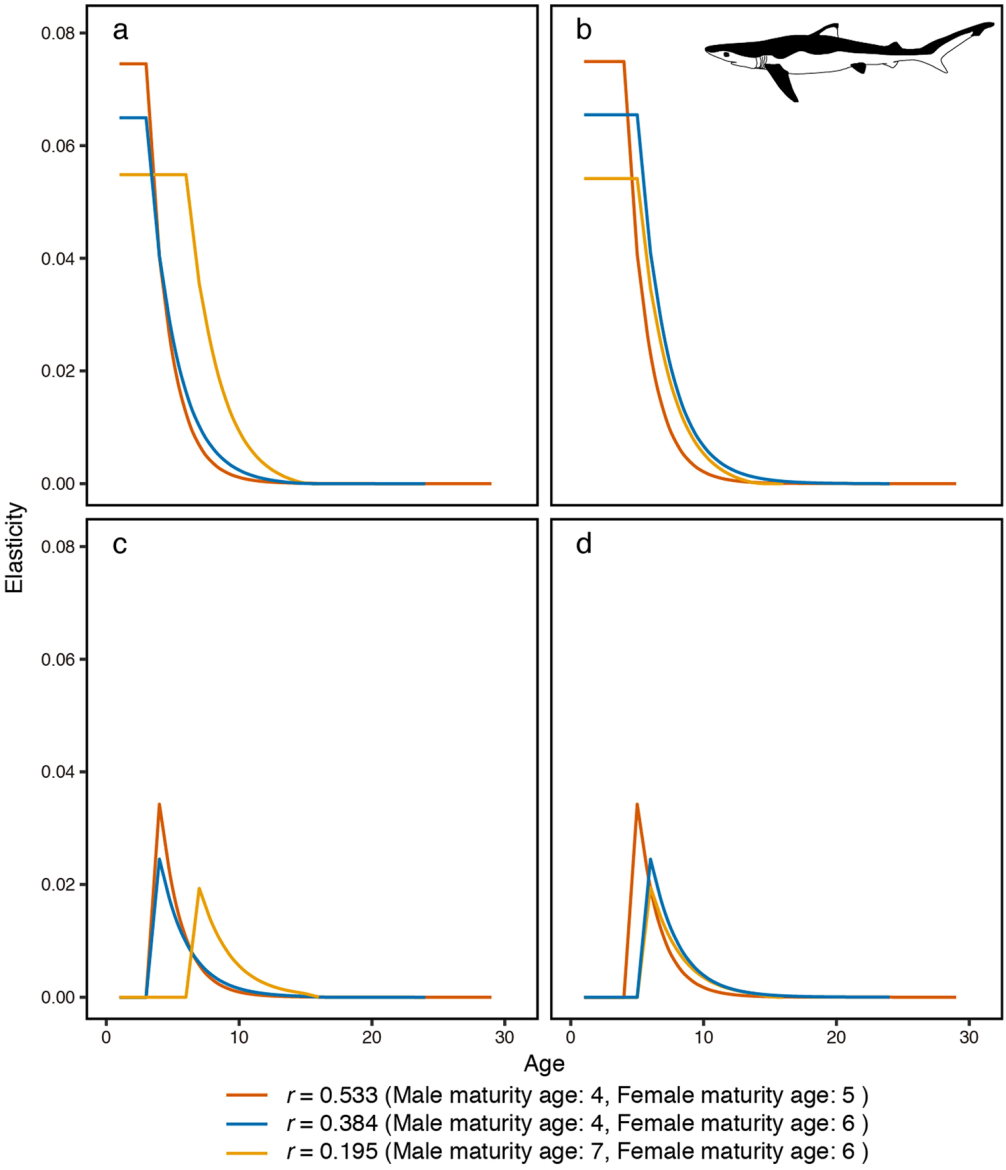
The elasticity of the population growth rate. Elasticity was estimated using several biological parameters of the blue shark (*Prionace glauca*) and was calculated using highest, median and lowest population growth rates. (**a**) The elasticity of the survival rate of male sharks. (**b**) The elasticity of the survival rate of female sharks. (**c**) The elasticity of male shark fecundity. (**d**) The elasticity of female shark fecundity.

**Figure 4:**
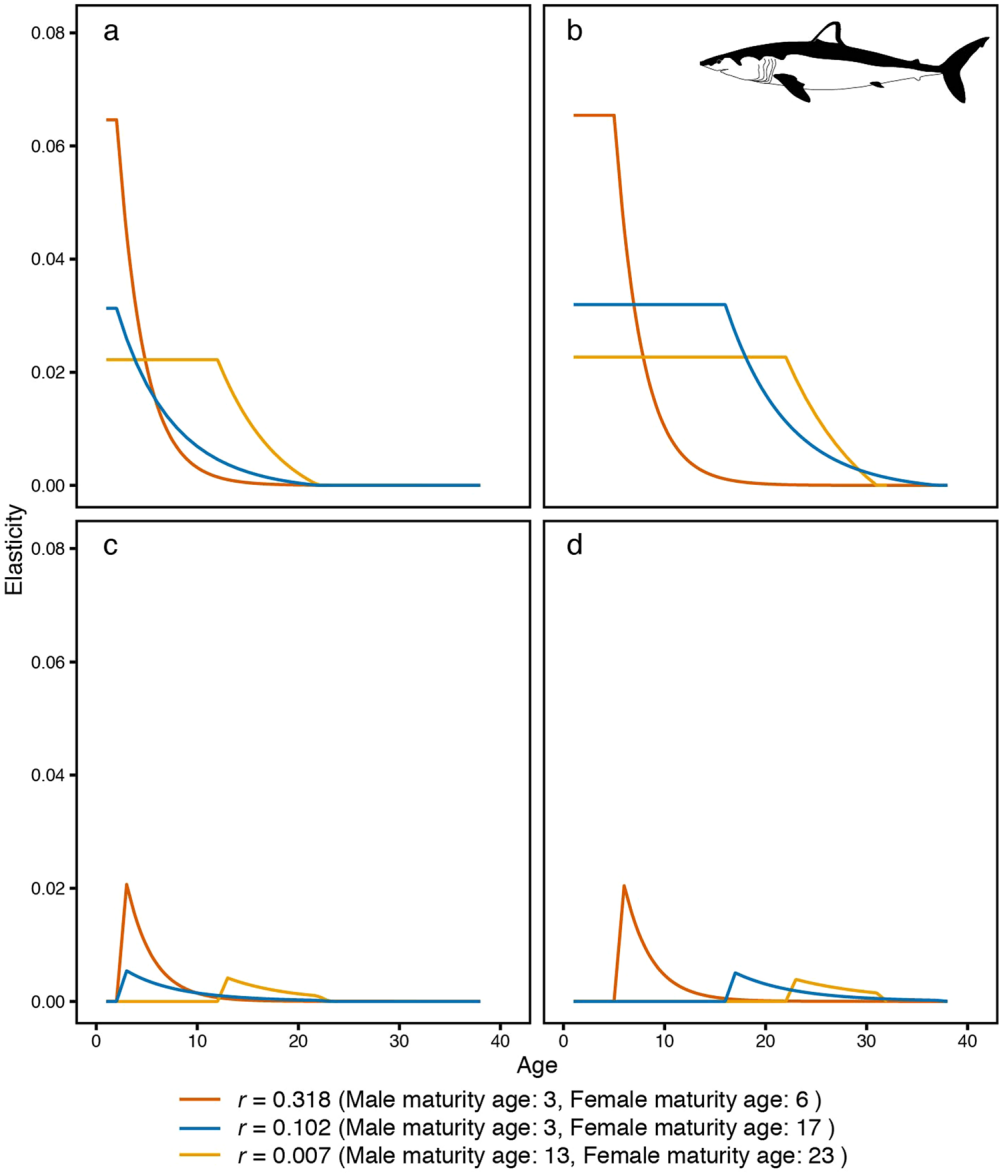
The elasticity of the population growth rate. Elasticity was estimated using several biological parameters of the shortfin mako shark (*Isurus oxyrinchus*) and was calculated using highest, median and lowest population growth rates. (**a**) The elasticity of the survival rate of male sharks. (**b**) The elasticity of the survival rate of female sharks. (**c**) The elasticity of male shark fecundity. (**d**) The elasticity of female shark fecundity.

We calculated the elasticity of the two-sex age-structured matrix model. The elements of this matrix were defined as a) the survival rate of male sharks; b) the survival rate of female sharks; c) the fecundity of male sharks and d) the fecundity of female sharks (Figs 3 and 4). In this analysis, we used three matrixes of the population growth rate, with highest, lowest and median values of estimated population growth rates.

For the blue shark, the elasticity of the survival rate by age was greater than the elasticity of fecundity by age for both sexes, with the elasticity of juvenile survival being particularly large (Fig. 3). The elasticity of the juvenile male survival rate by age was constant (0.055, 0.065 and 0.075), with corresponding population growth rates of 0.195, 0.384 and 0.533, respectively (Fig. 3a). The elasticity of the juvenile female survival rate showed a similar trend to that of the juvenile males, with values of 0.054, 0.065 and 0.075 (Fig. 3b). After maturation, the elasticity of the survival rate decreased with increasing age (Fig. 3a,b). In contrast, the elasticity of juvenile shark fecundity by age for both sexes was zero and was maximised at maturity (Fig. 3c,d). The peak elasticities of male fecundity were 0.019, 0.025 and 0.034, with population growth rates of 0.195, 0.384 and 0.533, respectively (Fig. 3c). The maximum elasticities of female fecundity by age were 0.020, 0.024 and 0.034, with population growth rates of 0.195, 0.384 and 0.533, respectively (Fig. 3d). After maturation, the elasticity of fecundity decreased with increasing age for both sexes (Fig. 3c,d). The total elasticity of the survival rates was inversely proportional to population growth rate, with values of 0.782 (*r* = 0.195), 0.739 (*r* = 0.384) and 0.701 (*r* = 0.533) (Fig. 3a,b). In contrast, the total elasticity of fecundity increased with increasing population growth rate, with values of 0.109 (*r* = 0.195), 0.130 (*r* = 0.384) and 0.149 (*r* = 0.533) (Fig. 3c,d).

For the shortfin mako shark, the elasticity of the survival rate by age was also greater than the elasticity of fecundity by age (Fig. 4). The elasticity of the survival rate by age for juvenile sharks showed a constant high value that decreased with increasing population growth rate (Fig. 4a,b). The elasticities of the survival rate of juvenile males by age were 0.022, 0.031 and 0.065, with population growth rates of 0.007, 0.102 and 0.318, respectively (Fig. 4a). The elasticities of juvenile females by age were 0.023, 0.032 and 0.065 (Fig. 4b). After maturation, the elasticity of the survival rate for both sexes decreased with increasing age (Fig. 4a,b). The elasticity of fecundity during the juvenile period was zero for both sexes (Fig. 4c,d). The elasticity of male fecundity was maximised at the maturation age, with values of 0.004, 0.005 and 0.021 (Fig. 4c). The elasticity of fecundity in females was also maximised at the maturation age, with values of 0.004, 0.005 and 0.020 (Fig. 4d). The total elasticity of the female survival rate was larger than that of the male survival rate and was inversely proportional to the increasing population growth rate (Fig. 4a,b). The total elasticities of the male survival rate were 0.338 (*r* = 0.007), 0.205 (*r* = 0.102) and 0.268 (*r* = 0.318) (Fig. 4a). The total elasticities of the female survival rate were 0.572 (*r* = 0.007), 0.669 (*r* = 0.102) and 0.472 (*r* = 0.318) (Fig. 4b). The total elasticity of fecundity was smaller than that of the survival rate, with values of 0.045 (*r* = 0.007), 0.063 (*r* = 0.102) and 0.130 (*r* = 0.318) (Fig. 4c,d).

### Sensitivity analysis

**Figure 5:**
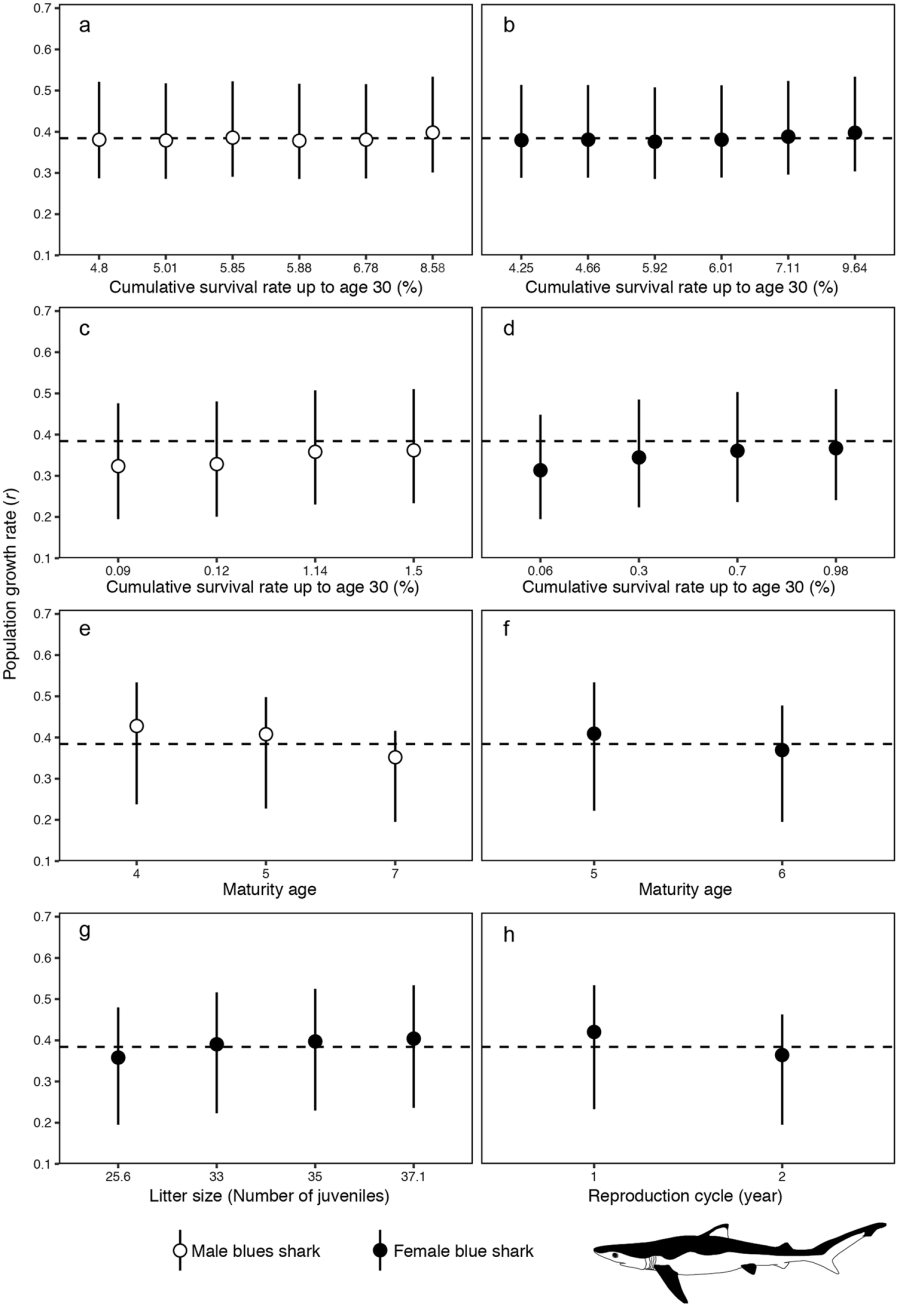
Estimated population growth rate (*r*) of the blue shark (*Prionace glauca*) using all combinations of biological parameters. Circles represent median values. Error bars represent minimum to maximum ranges. Dashed lines indicate the median of all estimated population growth rates. All estimated population growth rates were sorted by sex-dependent biological parameters (white circles: male; black circles: female). (**a**) Cumulative survival rate of male sharks up to age 30 (estimated by the Peterson and Wroblewski equation). (**b**) Cumulative survival rate of female sharks up to age 30 (estimated by the Peterson and Wroblewski equation). (**c**) Cumulative survival rate of male sharks up to age 30 (estimated by the Hoenig equation). (**d**) Cumulative survival rate of female sharks up to age 30 (estimated by the Hoenig equation). (**e**) Maturity age of male sharks. (**f**) Maturity age of female sharks. (**g**) Mean litter size. (**h**) Annual reproduction cycle.

We organised all estimated population growth rates by the different biological parameters for the blue shark (Fig. 5). The greatest effect on the estimated population growth rate was the male maturation age, and that difference was 0.076 (Fig. 5e). Female cumulative survival had the next largest influence on population growth rate (Fig. 5d). For males, when the male survival was assumed to be low, the lowest median population growth rate was estimated (*r* = 0.324) (Fig. 5c). In contrast for females, the highest median population growth rate was calculated using the one-year reproduction cycle (*r* = 0.420) (Fig. 5d). In this analysis, we used two estimation methods for survival rate, including the Peterson and Wroblewski equation (Fig. 5a,b) and the Hoenig equation (Fig. 5c,d). The median population growth rate tends to increase as the cumulative survival rate of male sharks, which is between 0.379 and 0.400, increases (Fig. 5a). The median population growth rate also increases with increasing cumulative female survival rate (0.376–0.400) (Fig. 5b). When the Hoenig equation was used, the median population growth rate was less than the median of all estimated population growth rates and increased with increasing cumulative survival rate for both sexes (male: 0.324–0.362; female: 0.313–0.367) (Fig. 5c,d). The younger maturation age was factored into the high median population growth rate for both sexes (male: 0.352–0.428; female: 0.369–0.409) (Fig. 5e,f). The larger the assumed litter size, the higher the estimated median population growth rate (0.368–0.404) (Fig. 5g). The median population growth rate in a one-year reproduction cycle was greater than that for a two-year reproduction cycle (0.420 and 0.364, respectively) (Fig. 5h).

**Figure 6:**
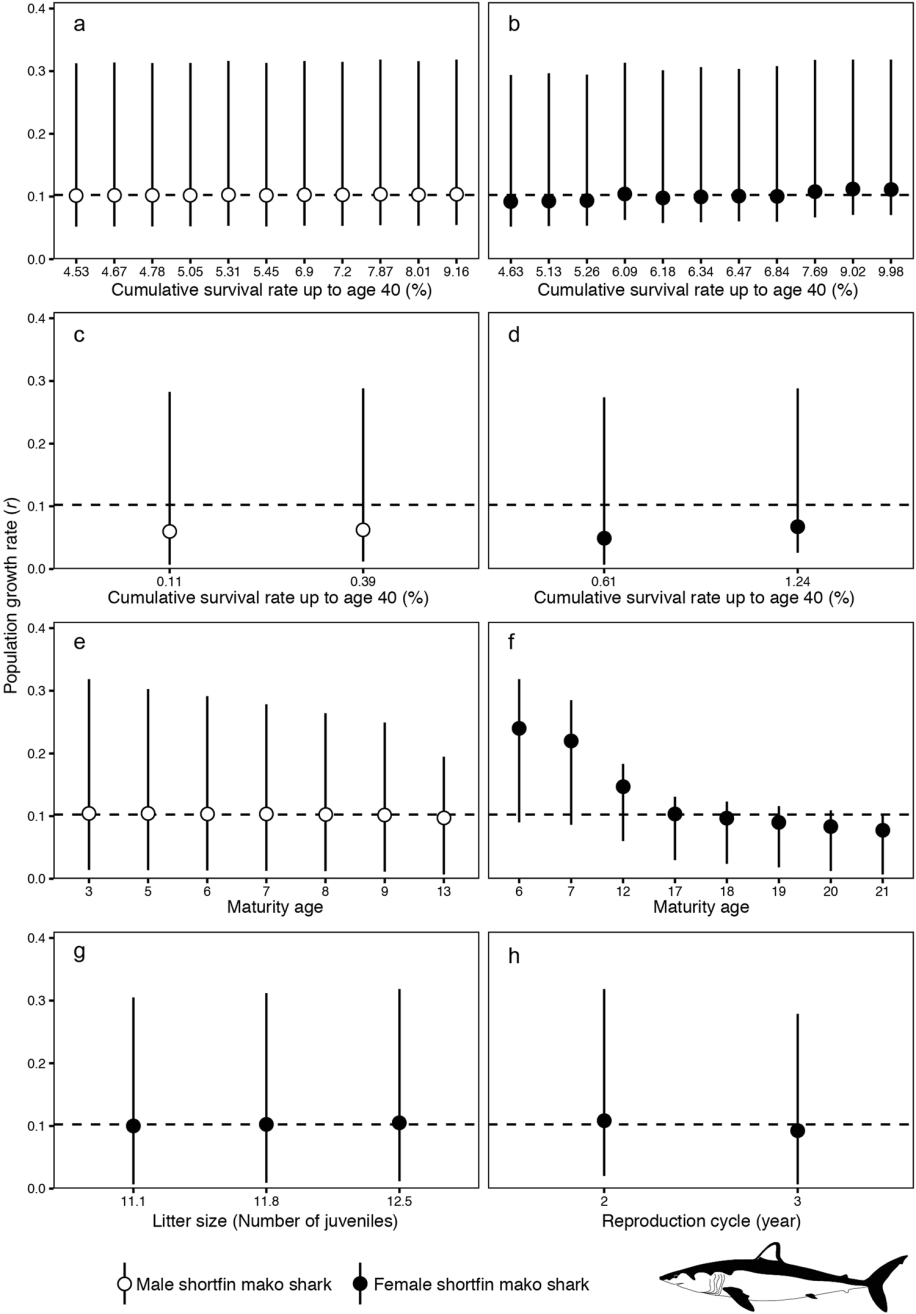
Estimated population growth rate (*r*) of the shortfin mako shark (*Isurus oxyrinchus*) using all combinations of biological parameters. Circles represent median values. Error bars represent minimum to maximum ranges. Dashed lines represent the medians of all estimated population growth rates. All estimated population growth rates were sorted by sex-dependent biological parameters (white circles: male, black circles: female). (**a**) Cumulative survival rate of male sharks up to age 40 (estimated by the Peterson and Wroblewski equation). (**b**) Cumulative survival rate of female sharks up to age 40 (estimated by the Peterson and Wroblewski equation). (**c**) Cumulative survival rate of male sharks up to age 40 (estimated by the Hoenig equation). (**d**) Cumulative survival rate of female sharks up to age 40 (estimated by the Hoenig equation). (**e**) Maturity age of male sharks. (**f**) Maturity age of female sharks. (**g**) Mean litter size. (**h**) Annual reproduction cycle.

All estimated population growth rates for the shortfin mako shark were organised by each biological parameter (Fig. 6). Female maturation age had the greatest influence on the estimated population growth rate. In particular, when the maturation age was assumed to be 6 or 7 years, the estimated population growth rates were considerably high (Fig. 6). When we applied the Peterson and Wroblewski equation to estimate the survival rates of male sharks, the median population growth rate changed slightly (0.102–0.104) (Fig. 6a). In contrast, the population growth rate showed an increasing trend in the high female shark survival rate, with a median value between 0.092 and 0.112 (Fig. 6b). We also used the Hoenig equation for estimating the survival rate estimation, which revealed that each median population growth rate was less than the median of all the estimated population growth rates (Fig. 6c,d). Cumulative survival rates, up to age 40, for male sharks were estimated between 0.11% and 0.39%, with median population growth rates of 0.060 and 0.063, respectively (Fig. 6c). Similarly, female survival rates, up to age 40, were estimated between 0.61% and 1.24%, with median population growth rates of 0.049 and 0.068, respectively (Fig. 6d). The median population growth rate slightly increased with decreasing male maturation age, with values between 0.097 and 0.105 (Fig. 6e). When younger female maturation ages were assumed, the median population growth rate increased (0.078–0.240) (Fig. 6f). A small effect of the average litter size on population growth rate was seen with values between 0.100 and 0.105 (Fig. 6g). Higher population growth rates were estimated under a two-year reproduction cycle (0.109) compared with a three-year reproduction cycle (0.092) (Fig. 6h).

## Discussion

We developed a two-sex age-structured matrix population model that reflects more realistic life cycles of blue and shortfin mako sharks to evaluate the effects of biological parameters on the population growth rate. To estimate appropriate population growth rates, we applied combinations of reported biological parameters to this matrix model (Tables S1 and S2). Consequently, we obtained several types of population growth rates with various biological parameter combinations; the median values and the ranges for both sharks were calculated (Table 1). These population growth rate values can be used as the prior distribution or the initial values for the Bayesian surplus production model (BSPM)^52, 53^, as well as the stock production model incorporating covariates (ASPIC)^54^, under the stock assessment scheme and ERA productivity index. Our results are useful for these assessments. For instance, the stock sizes of shortfin mako sharks in the Atlantic Ocean and blue sharks in the North Pacific Ocean were applied using the BSPM^25, 26^. The trend of these stock assessment results was based on the abundance index from the CPUE (catch per unit effort). However, it has been reported that the increasing tendency of CPUE is larger than the population growth rate for shortfin mako sharks^55^. Our findings suggest the possibility of higher population growth rates than previous studies (Table 1). Thus, this high population growth rate would improve future stock assessments of shortfin mako sharks. Furthermore, this CPUE would support the validation of our two-sex age-structured matrix model because the CPUE was statistically standardised and its data source was different from that of this study.

In the present study, by using elasticity analysis, we evaluated how biological parameters affect the population growth rate. As a result, high elasticity was shown for the juvenile survival rates of the blue and shortfin mako sharks (Figs 3 and 4). The impact of juvenile survival rate increased with decreasing population growth rates (Figs 3 and 4). These results indicate that the survival rate of juveniles plays an important role in short-term proportional changes in the population growth rate for both pelagic sharks. In particular, for shortfin mako sharks, the impact of the female survival rate was large (Fig. 4b). To clarify the biological parameter effect for both sharks in detail, we performed a sensitivity analysis (Figs 5 and 6). The age of maturity for male sharks had the greatest impact on the blue shark population growth rate (Fig. 5e); female survival rates had the second largest impact (Fig. 5d). One reason for this difference may be that the range of male maturity ages is larger than that for females.

For the shortfin mako shark, the maturity age of females had the greatest impact on population growth rate (Fig. 6f). The importance of juvenile shark survival has been indicated in previous studies as pelagic sharks are considered to have an extreme life history strategy, which includes a long-matured age^33, 39, 49^. Juvenile shark survival consists of maturity age and natural mortality by sex. In this study, we demonstrated the importance of sex differences in maturation age for estimating accurate population growth rates for both pelagic sharks. Thus, because of the assumption of the larger range of maturity age, the elasticity and estimated long-term population growth rate for shortfin mako sharks have greater variability than blue sharks.

The quality of data available on maturation age and survival rates depends on ageing validation techniques. For instance, maturity-length data were converted to maturity-age data using growth curves. The growth curve is estimated using individual ageing data. Natural mortality also depends on ageing validation because it is usually estimated by weight at age or longevity^56^. Thus, the precise ageing validation of pelagic sharks is a fundamental issue for calculating population growth rates. However, previous studies on growth curves for the shortfin mako shark show high variability (Fig. 2b). Usually, individual ageing data (length at age) are used for growth curve estimations, and individual ageing is addressed by vertebrae band-pair reading^40–43, 45, 57–62^. Band-pair reading includes several uncertainties, such as reading errors and differing reading methods. Therefore, one of the reasons for different growth curves may be variations in the band-pair reading method. In the past, band-pair reading of vertebrae for shortfin mako sharks consisted of three methods: one band-pair reading annually, two band-pair readings annually and two band-pair readings annually until five years of age^62^. Currently, only one band-pair reading annually is typically used. This reading method tends to provide a better estimation for slow growth, and high maturation ages have been determined under the slow growth assumption. As a result, the population growth rates of the shortfin mako shark currently estimated were relatively small (Table 1). These low values are consistent with our assumption of a high maturity age (Fig. 6f). However, some studies have attempted to estimate growth curves using other datasets such as tagging research data or length composition data^59, 63^. These studies have suggested earlier growth rather than the one band-pair reading growth curve (Fig. 2). To reflect this research, differences in the band-pair reading method were compared and have indicated an early maturation age for the shortfin mako shark^62^. We estimated population growth rates of the shortfin mako shark, including the early maturity age, accompanied with the first growth curve. Consequently, population growth rates of the shortfin mako shark in this study were greater than what has been reported previously (Table 1). For the blue shark, the difference in growth curves was relatively similar in the early period, with differences in maturation age slightly less than those for the shortfin mako shark (Fig. 1). These findings indicate that ageing validation of the blue shark is similar between previous studies. Consequently, the range of estimated population growth rates is relatively smaller than that for the shortfin mako shark. Reflecting this situation, we obtained similar results to previous studies (Table 1).

Despite various studies, there is a lack of knowledge regarding blue and shortfin mako shark biology. For instance, the possibility of a density-dependent effect on biological parameters that are changed by fishing exploitation has been reported^13^. However, the parameter effect of the density-dependent population has been found to be near equilibrium^64^. We did not consider a density-dependent relationship in the matrix population model because there is little to no information on density dependence. The mechanism of a density-dependent effect on pelagic shark biology or the population itself is complex. However, it is believed that biological information corresponds to populations that have been heavily exploited. Thus, the population growth rate that we computed should be relatively close to maximum values in theory. We used mean litter size for our analysis; actual ranges in litter size may be larger than our approximation because litter size varies individually. For the blue shark, a proportional relationship between shark length and litter size has been reported^65, 66^. Several research studies have reported differences in biological information by ocean (Tables S1 and S2); thus, ERA and stock assessments may need to reflect localised biological parameters. For the shortfin mako shark, the possibility that the mating mechanism has a large impact on the population growth rate has been reported^50^. We did not consider the mating system (e.g., monogamous, polyandrous, or polygynous) because information on the mating system of these sharks does not currently exist. Considering these biological uncertainties, the actual range of population growth rates may be larger than our assumption.

Additional studies of the biology of pelagic sharks are necessary, particularly for obtaining accurate growth curves as this uncertainty significantly affects population growth rates. Natural mortality is also important; in this study, we assumed two types of hypotheses for the mortality process (the Hoenig and Peterson and Wroblewski equations). Our results indicated that the Hoenig equation tends to estimate lower population growth rates than those estimated by the Peterson and Wroblewski equation (Figs 5 and 6). However, some studies have verified the use of these theoretical models for blue and shortfin mako sharks. Therefore, further consideration should be given to natural mortality estimates and their verification. For instance, total mortality can be estimated directly using tagging research results^67^. This information is useful for understanding realistic natural mortality rates.

The two-sex age-structured matrix model we developed could also assume other hypotheses of population dynamics, considering important factors such as age-sex biological features. Therefore, if the hypothesis of population dynamics changes, our model will still be useful for further research on biological parameters and their impact on population growth estimation. In addition, this methodology is useful for other species for which biological parameters are not well known.

## Methods

### Two-sex age-structured matrix population model

**Figure 7:**
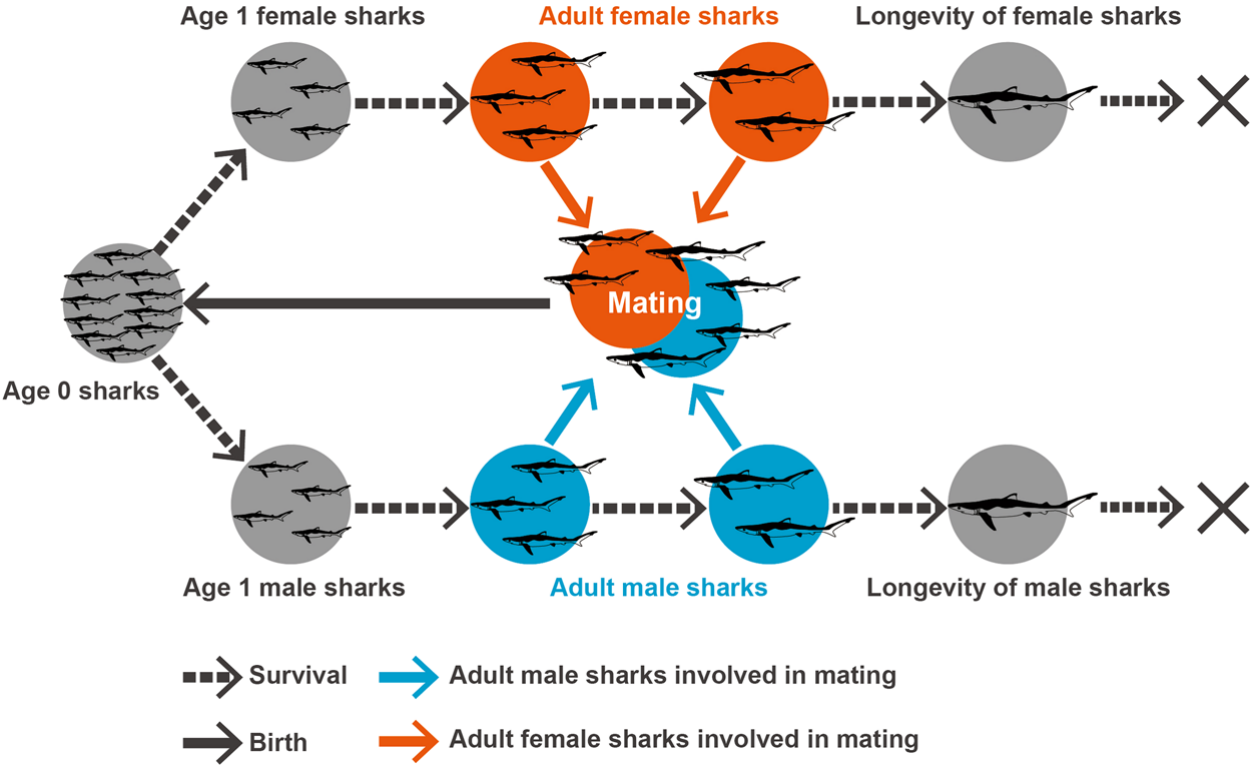
Life cycle of the blue and shortfin mako shark under the monogamy mating system.

The life history of large pelagic sharks shows sexual dimorphism, and we assumed a monogamous mating system in which the number of Age 0 sharks depends on the relative abundance of adult males and females (Fig. 7). To express sexual dimorphism in the life history of large pelagic sharks in the matrix population model, we constructed a two-sex age-structured matrix population model for blue and shortfin mako sharks: **N**_*t*+1_ = **A**_*t*_ **N**_*t*_. where **N**_*t*_ is a vector of population numbers for sharks at age *a* (1 ≤ *a* ≤ *a*_*max*_) in year *t* for both sex *s* (*s* = *m*, *f*), with elements of the vector being *n*_*t*,*a*,*s*_. **A**_*t*_ is the projection matrix in year *t* whose size defines the longevity of each sex and assumes a seasonal parturition^63, 65, 68^. The projection matrix **A**_*t*_ can be described as

1 $${{\bf{A}}}_{t}=(\begin{array}{ccccccccc}0 & {F}_{t,1,m} & \ldots  & {F}_{t,{a}_{max-1},m} & 0 & {F}_{t,1,f} & \ldots  & {F}_{t,{a}_{max-1},f} & 0\\ \rho {S}_{0} & 0 & \ldots  & 0 & 0 & 0 & \ldots  & 0 & 0\\ 0 & {S}_{1,m} & \ldots  & 0 & 0 & 0 & \ldots  & 0 & 0\\ \vdots  & \vdots  & \ddots  & \vdots  & \vdots  & \vdots  & \vdots  & \vdots  & \vdots \\ 0 & 0 & \ldots  & {S}_{{a}_{max-1},m} & 0 & 0 & \ldots  & 0 & 0\\ (1-\rho ){S}_{0} & 0 & \ldots  & 0 & 0 & 0 & \ldots  & 0 & 0\\ 0 & 0 & \ldots  & 0 & 0 & {S}_{1,f} & \ldots  & 0 & 0\\ \vdots  & \vdots  & \ldots  & \vdots  & \vdots  & \vdots  & \ddots  & \vdots  & \vdots \\ 0 & 0 & \ldots  & 0 & 0 & 0 & \ldots  & {S}_{{a}_{max-1},f} & 0\end{array}).$$

where *ρ* is the sex ratio, *S*_0_ is the survival rate under a no fishing condition for age 0 sharks, *S*_*a*,*s*_ is the survival rate under a no fishing condition for each age and sex, and *F*_*t*,__*a*,*s*_ is a fertility metric for each age and sex in year *t*. These matrix elements were calculated by several types of biological parameters, including litter size, maturation age, reproduction cycle, longevity, growth curve and weight-length relationship.

*S*_*a*,*s*_ is given by ${S}_{a,s}={e}^{-{M}_{a,s}}$ and *M*_*a*,*s*_ is the natural mortality at age by each sex. *S*_0_ is the calculated mean of natural mortality at age 0 for both sexes ${S}_{0}=\mathrm{0.5(}{e}^{-{M}_{\mathrm{0,}m}}+{e}^{-{M}_{\mathrm{0,}f}})$. Natural mortality for blue and shortfin mako sharks was estimated using several theoretical models^69–75^. These models were classified by four types of theories that associated the growth curve, weight at age, maturity and longevity parameters. We selected two equations to estimate the natural mortality of pelagic sharks; these methods have typically been used for shark studies^74–76^. Using another two type equations, we also estimated natural mortality^70^. However, neither pelagic shark could live to the maximum age that we supposed in the life cycle of sharks. Thus, we did not use these two theoretical models to maintain consistency with the population dynamics. (We estimated population growth rate using the natural mortality in Supplemental Information 3). The age-constant natural mortality equation that depends on longevity was given by ln (*M*_*a*,*s*_) = 0.941–0.873ln(*a*_*max*_)^74^. These coefficients have been assumed for whales and are typically used for large pelagic sharks^39, 47, 49, 50, 61^. Alternatively, age-different natural mortality was estimated using mean body weight at age by sex *W*_*a*,*s*_ with values ${M}_{a,s}=1.28{W}_{a,s}^{-0.25}$. *W*_*a*,*s*_ is given by the weight-length relationship ${W}_{a,s}={10}^{-3}\alpha {L}_{a,s}^{\beta }$, where *α* and *β* are coefficients to convert length to weight. *L*_*a*,*s*_ is the total length (for the blue shark) or precaudal length (for the shortfin mako shark) given by the growth curve from the von Bertalanffy or Gompertz equation. The growth curves of both sharks were reported by fork length (*FL*), total length (*TL*), and precaudal length (*PL*). The *TL* and *FL* data of the blue shark were converted to *PL*, because the weight-length relationship was assumed *PL* ^65^. To convert *TL* to *PL*, we used the transformation equation *PL* = 0.762*TL* − 2.505^77^. *FL* was changed by *PL* = 0.975*FL* − 0.395^78^. For the shortfin mako shark, we converted *FL* and *PL* to *TL* because the weight-length relationship was written in *TL* ^61^. Hence, *PL* was converted by *PL* = 0.84*TL* − 2.13^60^. *FL* was converted by *PL* = 0.894*FL*  + 2.192(male) and *PL* = 0.905*FL* + 1.345(female)^43^.

*F*_*t*,*a*,*s*_ is given by a nonlinear equation with *F*_*t*,*a*,*s*_ = *f*_*t*,*s*_ *γ*_*a*,*s*_ *S*_*a*,*s*_ *δ*, where *f*_*t*,*s*_ is a fecundity function, *γ*_*a*,*s*_ is the maturity rate by age and sex, and *δ* is the reproduction cycle. *f*_*t*,*s*_ is the equation of the relative number of births by individual derived from the relative population number of adult males or females *R*_*t*,*s*_ and litter size *k*:

2 $${f}_{t,s}=\{\begin{array}{cc}\frac{k{R}_{t,f}}{{R}_{t,m}+{R}_{t,f}} & s=m\\ \frac{k{R}_{t,m}}{{R}_{t,m}+{R}_{t,f}} & s=f\end{array}$$

and

3 $${R}_{t,s}=\sum _{a\mathrm{=1}}^{{a}_{max}}{\gamma }_{a,s}{n}_{t,a,s}\delta \mathrm{.}$$

To consider mating success, *f*_*t*,*s*_ describes the sex interaction^34, 49, 50^. *γ*_*a*,*s*_ is 0 or 1 given by the maturation age of both sexes (e.g., if the age of the shark exceeds 50% maturity age, *γ*_*a*,*s*_ = 1; else *γ*_*a*,*s*_ = 0). We assumed that male sharks reproduce every year (*δ* = 1). The reproduction cycle of the female shark was assumed to be one year (*δ* = 1), two years (*δ* = 0.5), or three years (*δ* = 0.33). An example of the two-sex age-structured matrix population models is described in the supplemental data (see Supplemental Data).

### Population growth rate

Using all considerable biological parameter combinations for both sharks, we calculated the population growth rates. Several studies for both pelagic sharks’ biology exist. To obtain the appropriate population growth rate, we performed biological parameter screening for both pelagic sharks (Tables S1 and S2). The population growth rates (*r*) were calculated using a maximum eigenvalue *λ* of the projection matrix **A**_*t*_. The stable projection matrix with the stable age distribution was used for all analysis, because the projection matrix and the eigenvalue will change by age distribution. The stable projection matrix was calculated 3,000 times by repeated multiplication using the projection matrix. In this calculation, we generated the initial conditions of the population vector given by uniform random numbers ${n}_{\mathrm{0,}a,s} \sim U\mathrm{(0,1)}$. All population growth rates were calculated using Mathematica version 10.4.1.0; a simple example written in R is available in the supplement (see Supplemental Information 3). There are several studies of population growth rates for both pelagic sharks^25, 46–51, 79, 80^. These estimated population growth rates were compared with our study results.

### Elasticity analysis

To identify essential elements of the population growth associated with the life history of pelagic sharks, we performed an elasticity analysis^81–83^. The elasticity quantifies the proportional change of *λ*, which is affected by the proportional change of individual matrix elements *a*_*ij*_. The elasticity of *λ* is given by ${e}_{ij}=\frac{{a}_{ij}}{\lambda }\frac{\partial \lambda }{\partial {a}_{ij}}=\frac{\partial log\lambda }{\partial log{a}_{ij}}$, where *e*_*ij*_ denotes the elasticity of row *i* and column *j*. ∂*λ*/∂*a*_*ij*_ is the sensitivity of *λ* against the change in individual matrix elements *a*_*ij*_. The matrix element sensitivity of *λ* was calculated by $\frac{\partial \lambda }{\partial {a}_{ij}}=\frac{{v}_{i}{w}_{j}}{\langle w,v\rangle }$, where *w* and *v* are the right- and left-hand vectors, and 〈*w*,*v*〉 denote the scalar product of these two vectors. In this analysis, we used three matrix models where population growth rates are the highest, median and lowest of all estimated matrix models.

### Sensitivity analysis

To clearly understand the actual effects of the different biological parameters, we performed a sensitivity analysis, which was extended to include all possible parameter combinations. All calculated population growth rates were sorted by the different types of biological parameters, including survival rates of males, survival rates of females, maturation age of males, maturation age of females, litter size and reproduction cycle. All sorted values included the calculated maximum, median and minimum values. Evaluating the effect of survival rates was more complex because they were estimated by two types of theoretical models used for the different biological parameters of longevity and the growth curve. Because we needed to define the differences between the different estimation methods for survival rates and the biological parameters, we used an index in which the total survival rate was up to 30 (for the blue shark) and 40 (for the shortfin mako shark) (*S*30_*s*_ and *S*40_*s*_, respectively) for each sex (e.g., $S{40}_{s}={\prod }_{a\mathrm{=1}}^{40}{S}_{a,s}$).

## Electronic supplementary material

Supplementary information

A example of two-sex age-structured matrix population model
