# Supplementary material for: Impact of biology knowledge on the conservation and management of large pelagic sharks
Source: Sci Rep. 2017 Sep 6;7:10619. doi: 10.1038/s41598-017-09427-3 (PMC5587552; doi:10.1038/s41598-017-09427-3)
Supplement: Supplementary file 1 — Supplementary information [file 41598_2017_9427_MOESM1_ESM.pdf]

Impact of biology knowledge on the conservation and management of large pelagic sharks

Hiroki Yokoi, Hirotaka Ijima, Seiji Ohshimo, and Kotaro Yokawa

Supplementary information 1: List of available biological parameters

Table S1. Summary of the biological parameters of the blue shark (*Prionace glauca*). Bold letters are used to estimate population growth rates.

Definition	Value / Equation	Covered area	Reference
Sex ratio	**0.5**	North Pacific	1
	**0.5**	North Pacific	2
	**0.5**	South Pacific	3
Litter size	1–62 (n=669, mean=**25.6**)	North Pacific	1
	25–43 (n=5068, mean=**37.1**)	Global	4
	9–64 (n=37, mode=33)	North Pacific	5
	13–68 (n=46, mean=**35**)	South Pacific	3
Maturity age	[a] Male: **4–5**, [a] Female: **5–6**	North Pacific	1
	[b] Male: 4–5, [b] Female: 5	North Pacific	6
	[a] Male: **7**, [a] Female: **6**	South Africa	7
Reproduction cycle (years)	**1**, **2**	North Pacific	8
Longevity (age)	[c,d] Male: **16**, **16.5**, [c,d] Female: **15**, **26.1**	North Atlantic	6
	[c] Male: **16**, [c] Female: 12	North Pacific	9
	[f] Male: **26.866**, **28.642**, [g] Female: **24.068**, **20.150**	North Pacific	10
	[c,e,g] All:15, 21.4, 26.6	South Atlantic	11
Growth curve (cm)	[h] $L_a = 423.0[1-e^{-0.11(a+1.035)}]$	North Atlantic	12
	[h] $L_a = 401.55[1-e^{-0.13(a+0.62)}]$	Mediterranean	13
	[h] $L_{a,\mathrm{m}} = \mathbf{295.3}[1-e^{\mathbf{-0.175}(a+\mathbf{1.113})}]$, [h] $L_{a,\mathrm{f}} = \mathbf{241.9}[1-e^{\mathbf{-0.251}(a+\mathbf{0.795})}]$	North Pacific	14
	[h] $L_{a,\mathrm{m}} = \mathbf{369.0}[1-e^{\mathbf{-0.10}(a+\mathbf{1.38})}]$, [h] $L_{a,\mathrm{f}} = \mathbf{304.0}[1-e^{\mathbf{-0.16}(a+\mathbf{1.01})}]$	North Pacific	15
	[i] $L_{a,\mathrm{m}} = \mathbf{289.7}[1-e^{\mathbf{-0.129}(a+\mathbf{0.756})}]$, [i] $L_{a,\mathrm{f}} = \mathbf{243.3}[1-e^{\mathbf{-0.144}(a+\mathbf{0.849})}]$	North Pacific	1
	[j] $L_{a,\mathrm{m}} = \mathbf{282.3}[1-e^{\mathbf{-0.18}(a+\mathbf{1.35})}]$, [j] $L_{a,\mathrm{f}} = \mathbf{310.8}[1-e^{\mathbf{-0.13}(a+\mathbf{1.77})}]$	North Atlantic	16
	[h] $L_a = 352.1[1-e^{-0.157(a+1.01)}]$	South Atlantic	17
	[h] $L_{a,\mathrm{m}} = \mathbf{299.85}[1-e^{\mathbf{-0.10}(a+\mathbf{2.44})}]$, [h] $L_{a,\mathrm{f}} = \mathbf{237.5}[1-e^{\mathbf{-0.15}(a+\mathbf{2.15})}]$	North Pacific	9
	[h] $L_{a,\mathrm{m}} = \mathbf{294.6}[1-e^{\mathbf{-0.14}(a+\mathbf{1.30})}]$, [h] $L_{a,\mathrm{f}} = \mathbf{334.7}[1-e^{\mathbf{-0.11}(a+\mathbf{2.19})}]$	South Africa	7
	[h] $L_a = 352.1[1-e^{-0.13(a+1.31)}]$	South Atlantic	11
Weight-length relationship (kg)	[k] $W_{a,\mathrm{m}} = 0.392\times10^{-6}\, L_{a,\mathrm{m}}^{3.41}$, [k] $W_{a,\mathrm{f}} = 0.131\times10^{-5}\, L_{a,\mathrm{f}}^{3.2}$	North Atlantic	12
	[k] $W_a = 2.57\times10^{-5}\, L_a^{3.05}$	North Pacific	18
	[l] $W_{a,\mathrm{f}} = \mathbf{5.388\times10^{-6}}\, L_{a,\mathrm{m}}^{\mathbf{3.102}}$, [l] $W_{a,\mathrm{m}} = \mathbf{3.293\times10^{-6}}\, L_{a,\mathrm{f}}^{\mathbf{3.225}}$	North Pacific	1

[a] 50% maturity age. [b] Full maturity age. [c] Maximum observed age from vertebral band counts. [d] Age at L_∞ from the von Bertalanffy growth equation. [e] Theoretical longevity[19]. [f] Theoretical longevity[20] using von the Bertalanffy growth equation[1,21]. [g] Theoretical longevity[22]. [h] Total length at age. [i] Precaudal length at age. [j] Fork length at age. [k] Weight and total length relationship. [l] Weight and precaudal length relationship.

Table S2. Summary of the biological parameter of shortfin mako shark (*Isurus oxyrinchus*). Bold letters were used to estimate the population growth rate.

Definition	Value / Equation	Covered area	Reference
Sex ratio	**0.5** (In birth)	North Pacific	23
	**0.5** (Under 30kg samples)	North Pacific	24
	**0.5** (In birth)	North Pacific	25
Litter size	[a] 4–18 (n=8)	Global	26
	[a] 4–27.5 (n=30, mean=**12.5**)	Global	27
	4 (n=2, mean=4)	South Pacific	28
	4–15 (n=22, mean=**11.1**)	North Pacific	23
	8–17 (n=9, mean=**11.8**)	North Pacific	25
	9–14 (n=3, mean=11.7)	Indian Ocean	29
Maturity age	Male: **8**, Female: **18**	North Atlantic	30
	Male: **7**–**9**, Female: **19**–**21**	South Pacific	31
	Male: **13**, Female: **19**	North Pacific	24
	Male: **5**, Female: **17**	North Pacific	25
	Male: **3**, **6**, Female: **6**, >**7**, >**12**	South Atlantic	32
Reproduction	**2** or **3**	Global	27
cycle (years)	**3**	North Pacific	23
Longevity (age)	[b] All: 45	North Pacific	14
	[b] All: 28	Global	33
	[c] Female: 24	Atlantic	34
	[c] Male: 9, [c] Female: 18	North Pacific	35
	[b,c] Male: 21, **29**, [b,c] Female: **38**, **32**	North Atlantic	30
	[d] Female: 31	North Atlantic	36
	[c] Male: 29, [c] Female: 28	South Pacific	31
	[c] Male: 14, [c] Female: 20	North Pacific	37
	[c] All: 25	South Pacific	38
	[c,e] Male:23.6, 31, [c,e] Female:30.6, 41	North Pacific	24
	[b] Male: 16, 19, **23**, Female 19, 24, 28	South Atlantic	32
Growth	[f] $L_a = 321.0[1\text{-}e^{-0.072(a+3.75)}]$	North Pacific	14
curve (cm)	[f] $L_a = 411.0[1\text{-}e^{-0.05(a+4.7)}]$	North Pacific	35
	K: 0.19, 0.17 L_∞: 231.03, 252.12	North Pacific	39
	[g] $L_{a,\mathrm{m}} = \mathbf{302}[1\text{-}e^{\mathbf{-0.266}(a+1)}]$, [g] $L_{a,\mathrm{f}} = \mathbf{345}[1\text{-}e^{\mathbf{-0.203}(a+1)}]$	North Atlantic	40
	[g] $L_{a,\mathrm{m}} = \mathbf{302.3}[1\text{-}e^{\mathbf{-0.052}(a+\mathbf{9})}]$, [g] $L_{a,\mathrm{f}} = \mathbf{820.1}[1\text{-}e^{\mathbf{-0.013}(a+\mathbf{11.3})}]$	South Pacific	31
	[g] $L_{a,\mathrm{m}} = \mathbf{253.3\text{-}181.7}e^{\mathbf{-0.125}a}$, [g] $L_{a,\mathrm{f}} = \mathbf{88.4}e^{\mathbf{1.42}[1-e^{-(\mathbf{0.087}a)}]}$	North Atlantic	30
	[h] $L_{a,\mathrm{m}} = \mathbf{60}+\mathbf{171.3}(1\text{-}e^{\mathbf{-0.156}a})$, [h] $L_{a,\mathrm{f}} = \mathbf{60}+\mathbf{248.6}(1\text{-}e^{\mathbf{-0.090}a})$	North Pacific	37
	[f] $L_{a,\mathrm{m}} = \mathbf{332.1}[1\text{-}e^{\mathbf{-0.056}(a+\mathbf{6.08})}]$, [f] $L_{a,\mathrm{f}} = \mathbf{413.8\text{-}339.8}e^{\mathbf{-0.05}a}$	North Pacific	24
	[f] $L_{a,\mathrm{m}} = \mathbf{296.60}[1\text{-}e^{\mathbf{-0.087}(a+\mathbf{3.58})}]$, [f] $L_{a,\mathrm{f}} = \mathbf{325.29}[1\text{-}e^{\mathbf{-0.076}(a+\mathbf{3.18})}]$	South Pacific	38

Table S2 Continued.

Definition	Value / Equation	Covered area	Reference
	$^{h}L_{a,\mathrm{m}} = \mathbf{274.4\text{-}214.4}e^{\mathbf{-0.19}a}$, $^{h}L_{a,\mathrm{f}} = \mathbf{239.4\text{-}179.4}e^{\mathbf{-0.25}a}$	North Pacific	41
	$^{g}L_{a,\mathrm{m}} = \mathbf{580}[1\text{-}e^{\mathbf{-0.021}(a\mathbf{+7.52})}]$, $^{g}L_{a,\mathrm{f}} = \mathbf{416}[1\text{-}e^{\mathbf{-0.035}(a\mathbf{+6.18})}]$	South Atlantic	42
	$^{g}L_{a,\mathrm{m}} = \mathbf{328.74}[1\text{-}e^{\mathbf{-0.08}(a\mathbf{+4.47})}]$, $^{g}L_{a,\mathrm{f}} = \mathbf{407.65}[1\text{-}e^{\mathbf{-0.04}(a\mathbf{+7.08})}]$	South Atlantic	32
	$^{g}L_{a,\mathrm{m}} = \mathbf{340.2}[1\text{-}e^{\mathbf{-0.14}(a\mathbf{+2.75})}]$, $^{g}L_{a,\mathrm{f}} = \mathbf{441.64}[1\text{-}e^{\mathbf{-0.07}(a\mathbf{+3.98})}]$	South Atlantic	32
	$^{g}L_{a,\mathrm{m}} = \mathbf{291.57}[1\text{-}e^{\mathbf{-0.2}\,(a\mathbf{+2.38})}]$, $^{g}L_{a,\mathrm{f}} = \mathbf{309.79}[1\text{-}e^{\mathbf{-0.13}(a\mathbf{+3.27})}]$	South Atlantic	32
Weight-length	$^{i}W_{a} = 5.2432\times10^{-6}\,L_{a}^{3.1407}$	North Atlantic	43
relationship (kg)	$^{j}W_{a} = 1.1\times10^{-5}\,L_{a}^{2.95}$	North Pacific	23
	$^{j}W_{a,\mathrm{f}} = \mathbf{1.9\times10^{-5}}\,L_{a,\mathrm{m}}^{\mathbf{2.8433}}$, $^{j}W_{a,\mathrm{f}} = \mathbf{1.6\times10^{-5}}\,L_{a,\mathrm{m}}^{\mathbf{2.867}}$	North Pacific	24

[a] Summarize several information of litter size. [b] Estimated longevity by the von Bertalanffy growth equation. [c] Maximum observed age from vertebral band counts. [d] Maximum observed age from bomb radiocarbon chronologies. [e] Maximum observed age × 1.3. [f] Total length at age. [g] Fork length at age. [h] Precaudal length at age. [i] Weight and fork length relationship. [j] Weight and total length relationship.

Supplementary information 2: Population growth rates estimated by alternative natural mortality.

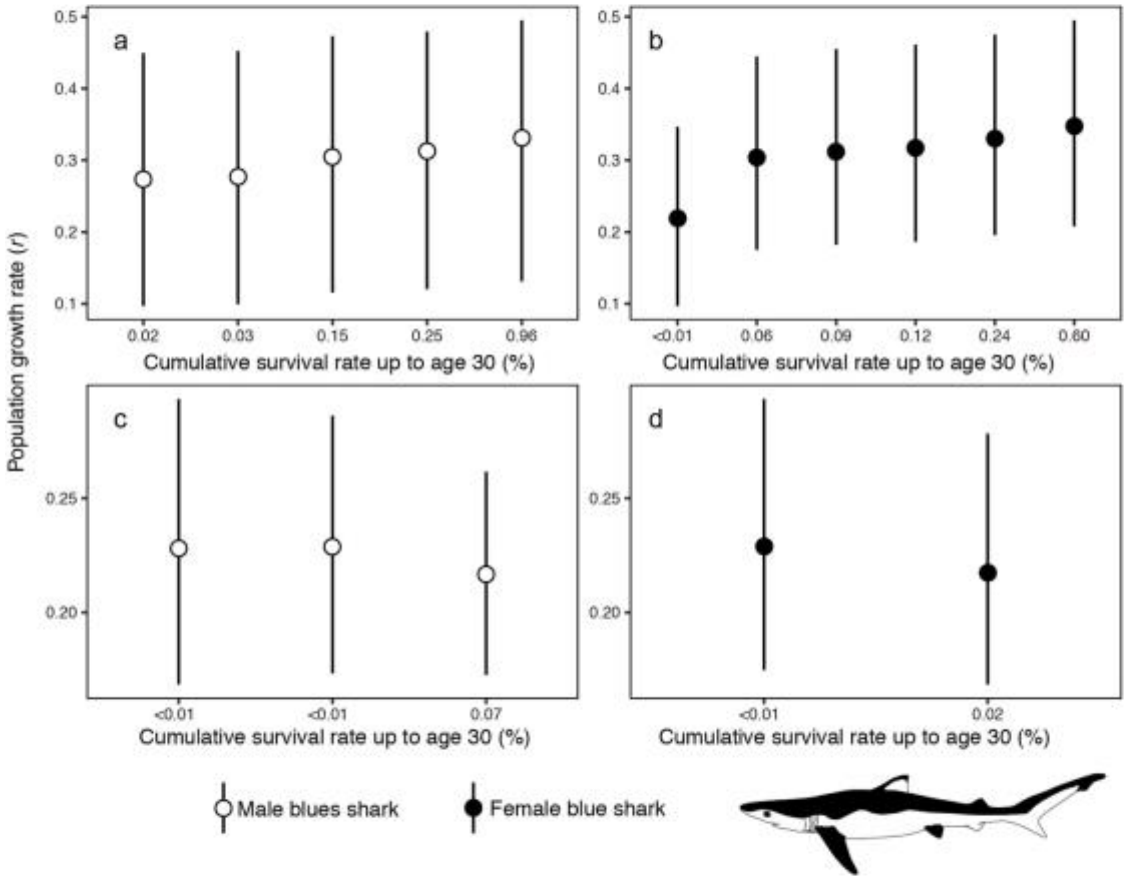

Figure S1. Estimated population growth rate *r* of the blue shark (*Prionace glauca*) using alternative natural mortalities[44]. Circles represent median values. Error bars represent minimum to maximum ranges. All estimated population growth rates were sorted by sex-dependent biological parameters (white circles: male; black circles: female). a) Cumulative survival rate of male sharks up to age 30 (estimated by the parameter of growth curve *k*). b) Cumulative survival rate of female sharks up to age 30 (estimated by growth parameter *k*). c) Cumulative survival rate of male sharks up to age 30 (estimated by maturity age). d) Cumulative survival rate of female sharks up to age 30 (estimated by maturity age).

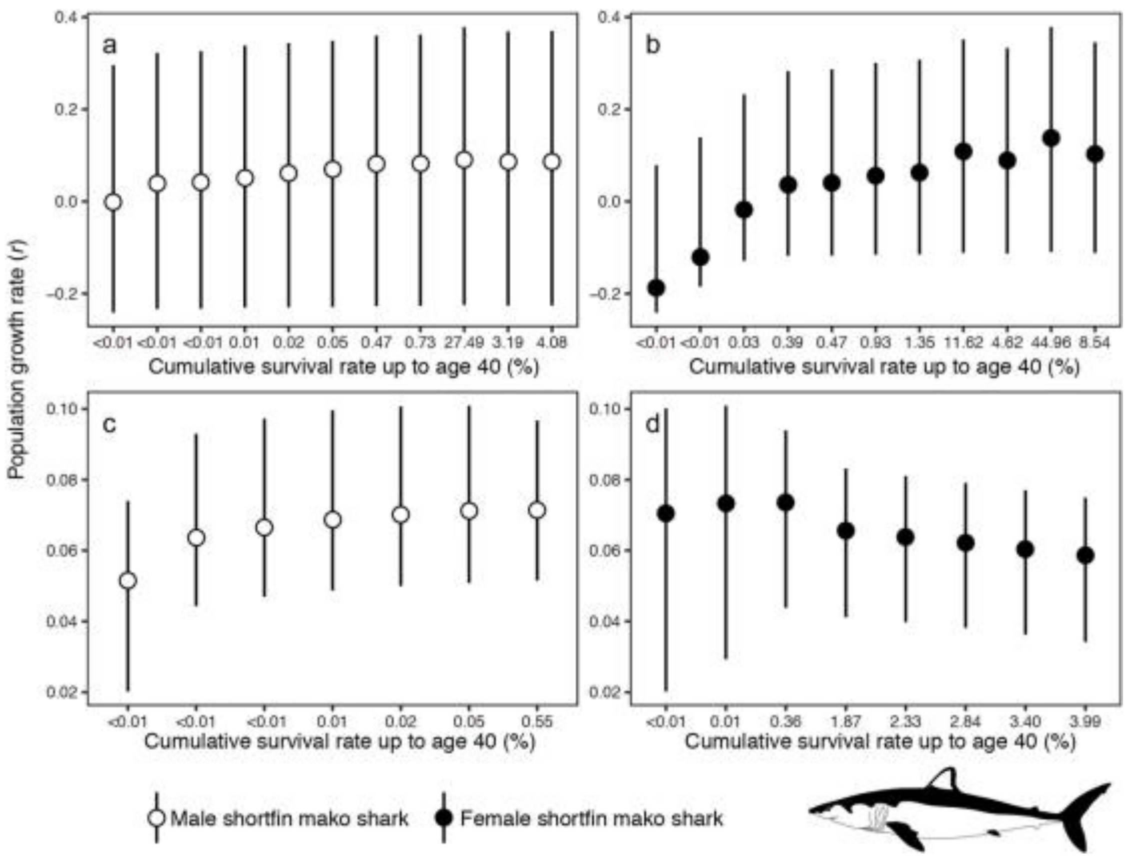

Figure S2. Estimated population growth rate *r* of the shortfin mako shark (*Isurus oxyrinchus*) using alternative natural mortalities[44]. Circles represent median values. Error bars represent minimum to maximum ranges. All estimated population growth rates were sorted by sex-dependent biological parameters (white circles: male, black circles: female). a) Cumulative survival rate of male sharks up to age 40 (estimated by the parameter of growth curve). b) Cumulative survival rate of female sharks up to age 40 (estimated by the parameter of growth curve). c) Cumulative survival rate of male sharks up to age 40 (estimated by maturity age). d) Cumulative survival rate of female sharks up to age 40 (estimated by maturity age).

Supplementary information 3: Simple example for calculating population growth rate

R script of the Blue shark

Biological parameters corresponded to supplemental_data.xlsx.

```
# Parameters--------------------------------------------------------------------------------------------------------------------------

# Longevity of male shark
long_m = 17

# Longevity of female shark
long_f = 21

# Survival rate of age 0 shark (Male and Female)
s_0 = 0.391581151

# Survival rate of male shark
s_m = c(0.834331185, 0.867872344, 0.88615173, 0.897594563, 0.905359817, 0.91091273, 0.915030454,
0.918165398, 0.920599639, 0.922518564, 0.9240492, 0.925281553, 0.92628118, 0.927096924, 0.927765872,
0.928316636, 0)

# Survival rate of female shark
s_f = c(0.861727024, 0.885356998, 0.899798628, 0.909515666, 0.916468953, 0.921661023, 0.925660109,
0.928813362, 0.931345349, 0.933408041, 0.935108101, 0.936522679, 0.937709009, 0.938710462, 0.939560518,
0.940285431, 0.940906073, 0.941439239, 0.941898585, 0.942295316, 0)

# Maturity at age of male shark
mat_m = c(0, 0, 0, 1, 1, 1, 1, 1, 1, 1, 1, 1, 1, 1, 1, 1, 1)

# Maturity at age of female shark
mat_f = c(0, 0, 0, 0, 0, 1, 1, 1, 1, 1, 1, 1, 1, 1, 1, 1, 1, 1, 1, 1, 1)

# Reproduction cycle of male shark
delta_m = 1

# Reproduction cycle of female shark (2 years)
delta_f = 0.5
```

```
#Litter size
k = 33

# Two sex age-structured Matrix model---------------------------------------------------------------------------------------------------

N = list()

# Set the initial population vector

N[[1]] = runif(long_f + long_m + 1) # Add age 0 shark

# 3000 times iteration

for (i in 1:3000) {

    # Calculate scaled population vector
    N[[i]] = N[[i]] / sum(N[[i]])

    # Calculate adult shark
    R_m = sum(mat_m * N[[i]][2:(long_m + 1)] *delta_m)
    R_f = sum(mat_f * N[[i]][(long_m + 2) : (long_f + long_m + 1)] * delta_f)

    # Calculate fecundity
    F_m = k * R_f / (R_f + R_m)
    F_f = k * R_m / (R_f + R_m)

    # Make the two sex age-structured matrix model
    A = matrix(0, nrow = long_f + long_m + 1, ncol = long_f + long_m + 1)

    A[1,] = c(0, F_m * mat_m * s_m * delta_m, F_f * mat_f * s_f * delta_f)
    A[2, 1] = s_0
    A[(long_m + 2), 1] = s_0

    S = c(s_m[-long_m], 0, s_f[-long_f])
    length_S = length(S)
```

```
        for (j in 1:length_S) {
                A[j+2, j+1] = S[j]
        }

        # Calculate next step population vector
        N[[i+1]] = A %*% N[[i]]

}

# Calculate population growth rate-----------------------------------------------------------------------------------------------------

r = log(eigen(A)$values[1])
```

R script of shortfin mako shark

Biological parameter was corresponded to supplemental_data.xlsx.

```
# Parameters------------------------------------------------------------------------------------------------------------------------------------------

# Longevity of male shark
long_m = 23

# Longevity of female shark
long_f = 38

# Survival rate of age 0 shark (Male and Female)
s_0 = 0.424527997

# Survival rate of male shark
s_m = c(0.878206651, 0.892725525, 0.902507082, 0.909528408, 0.914791525, 0.918862146, 0.922085424,
0.924684658, 0.926811112, 0.92857111, 0.930041701, 0.931280158, 0.932329975, 0.933224803, 0.933991089,
0.93464991, 0.935218264, 0.935710007, 0.936136537, 0.936507308, 0.936830218, 0.937111905, 0)

# Survival rate of female shark
s_f = c(0.866559031, 0.881659038, 0.89244894, 0.900557658, 0.906876001, 0.911935013, 0.916072258,
0.919513254, 0.922414777, 0.924889411, 0.927020232, 0.928869964, 0.930486906, 0.931908892, 0.933166005,
0.934282482, 0.935278083, 0.936169085, 0.936969028, 0.937689266, 0.938339397, 0.938927593, 0.939460855,
0.939945215, 0.940385902, 0.940787467, 0.941153893, 0.941488678, 0.941794907, 0.942075312, 0.942332316,
```

```
0.94256808, 0.942784534, 0.942983407, 0.943166249, 0.943334458, 0.943489294, 0)

# Maturity at age of male shark
mat_m = c(0, 0, 1, 1, 1, 1, 1, 1, 1, 1, 1, 1, 1, 1, 1, 1, 1, 1, 1, 1, 1, 1, 1)

# Maturity at age of female shark
mat_f = c(0, 0, 0, 0, 0, 0, 0, 0, 0, 0, 0, 0, 0, 0, 0, 1, 1, 1, 1, 1, 1, 1, 1, 1, 1, 1, 1, 1, 1, 1, 1, 1, 1, 1, 1, 1, 1)

# Reproduction cycle of male shark
delta_m = 1

# Reproduction cycle of female shark (2 years)
delta_f = 0.5

#Litter size
k = 11.1

# Two sex age-structured Matrix model-----------------------------------------------------------------------------------------------

N = list()

# Set the initial population vector

N[[1]] = runif(long_f + long_m + 1) # Add age 0 shark

# 3000 times iteration

for (i in 1:3000) {

    # Calculate scaled population vector
    N[[i]] = N[[i]] / sum(N[[i]])

    # Calculate adult shark
    R_m = sum(mat_m * N[[i]][2:(long_m + 1)] *delta_m)
    R_f = sum(mat_f * N[[i]][(long_m + 2) : (long_f + long_m + 1)] * delta_f)
```

```
    # Calculate fecundity
    F_m = k * R_f / (R_f + R_m)
    F_f = k * R_m / (R_f + R_m)

    # Make the two sex age-structured matrix model
    A = matrix(0, nrow = long_f + long_m + 1, ncol = long_f + long_m + 1)

    A[1,] = c(0, F_m * mat_m * s_m * delta_m, F_f * mat_f * s_f * delta_f)
    A[2, 1] = s_0
    A[(long_m + 2), 1] = s_0

    S = c(s_m[-long_m], 0, s_f[-long_f])
    length_S = length(S)

    for (j in 1:length_S) {
        A[j+2, j+1] = S[j]
    }

    # Calculate next step population vector
    N[[i+1]] = A %*% N[[i]]

}

# Calculate population growth rate----------------------------------------------------------------------------------------------------

r = log(eigen(A)$values[1])
```

References

1. Nakano, H. Age, reproduction and migration of blue shark (*Prionace glauca*) in the North Pacific Ocean. *Bull. Natl. Res. Inst. Far Seas Fish.* **31,** 141–256 (1994).
2. Castro, J. & Mejuto, J. Reproductive parameters of blue shark, *Prionace glauca*, and other sharks in the Gulf of Guinea. *Mar. Freshw. Res.* **46,** 967 (1995).
3. Zhu, J., Dai, X., Xu, L., Chen, X. & Chen, Y. Reproductive biology of female blue shark *Prionace glauca* in the southeastern Pacific Ocean. *Environ. Biol. Fishes* **91,** 95–102 (2011).
4. Mejuto, J. & García-Cortés, B. Reproductive and distribution parameters of the blue shark *Prionace glauca*, on the basis of on-board observations at sea in the Atlantic, Indian and Pacific oceans. *Collect. Vol. Sci. Pap. ICCAT* **58,** 951–973 (2005).
5. Fernandez, M. C., Galvan-Magana, F. & Vázquez, B. P. C. Reproductive biology of the blue shark *Prionace glauca* (Chondrichthyes: Carcharhinidae) off Baja California Sur, México. *aqua Int. J. Ichthyol.* **16,** 1–10 (2010).
6. Skomal, G. B. & Natanson, L. J. Age and growth of the blue shark (*Prionace glauca*) in the North Atlantic Ocean. *Collect. Vol. Sci. Pap.* **54,** 1212–1230 (2002).
7. Jolly, K., Silva, C. da & Attwood, C. Age, growth and reproductive biology of the blue shark *Prionace glauca* in South African waters. *African J. Mar. Sci.* **35,** 99–109 (2013).
8. Fujinami, Y., Semba, Y., Okamoto, H., Ohshimo, S. & Tanaka, S. Reproductive biology of the blue shark (*Prionace glauca*) in the western North Pacific Ocean. *Mar. Freshw. Res.* (2017). doi:10.1071/MF16101
9. Blanco-Parra, M. D. P., Galvan-Magana, F., Marquez-Farias, F., Galván-Magaña, F. & Márquez-Farías, F. Age and growth of the blue shark, Prionace glauca Linnaeus, 1758, in the Northwest coast off Mexico. *Rev. Biol. Mar. Oceanogr.* **43,** 513–520 (2008).
10. Rice, J. & Semba, Y. *Age and Sex Specific Natural Mortality of the Blue Shark (Prionace glauca) in the North Pacific Ocean*. (2014).
11. Hsu, H., Lyu, G., Joung, S. & Liu, K. Age and growth of the blue Shark (*Prionace glauca*) in the South Atlantic ocean. *Collect. Vol. Sci. Pap.* **71,** 2573–2584 (2015).
12. Stevens, J. D. Vertebral rings as a means of age determination in the blue shark (*Prionace glauca L.*). *J. Mar. Biol. Assoc. United Kingdom* **55,** 657–665 (1975).
13. Megalofonou, P., Damalas, D. & de Metrio, G. Biological characteristics of blue shark, *Prionace glauca*, in the Mediterranean Sea. *J. Mar. Biol. Assoc. United Kingdom* **89,** 1233–1242 (2009).
14. Cailliet, G. M. & Bedford, D. W. The biology of three pelagic sharks from California waters and their emerging fisheries: a review. *Calif. Coop. Ocean. Fish. Investig. Rep.* **24,** 57–69 (1983).
15. Tanaka, S., Cailliet, G. M. & Yudin, K. G. Differences in growth of the blue shark, *Prionace glauca*: technique or population? *NOAA Tech. Rep. NMFS* **90,** 177–187 (1990).
16. Skomal, G. & Natanson, L. Age and growth of the blue shark (*Prionace glauca*) in the North Atlantic Ocean. *Fish. Bull.* **101,** 627–639 (2003).
17. Lessa, R., Santana, F. M. & Hazin, F. H. Age and growth of the blue shark *Prionace glauca* (Linnaeus, 1758) off northeastern Brazil. *Fish. Res.* **66,** 19–30 (2004).
18. Harvey, J. T. Food habits, seasonal abundance, size, and sex of the blue shark, *Prionace glauca*, in Monterey Bay, California. *Calif. Fish Game* **75,** 33–44 (1989).
19. Fabens, A. J. Properties and fitting of the Von Bertalanffy growth curve. *Growth* **29,** 265–89 (1965).
20. Cailliet, G., Mollet, H., Pittenger, G., Bedford, D. & Natanson, L. Growth and demography of the Pacific Angle Shark (Squatina californica), based upon tag returns off California. *Mar. Freshw. Res.* **43,** 1313 (1992).
21. Hsu, H., Lyu, G., Joung, S. & Liu, K. *Age and growth of the blue shark, Prionace Glauca, in the central and south Pacific*. (2012).
22. Taylor, C. C. Cod Growth and Temperature. *ICES J. Mar. Sci.* **23,** 366–370 (1958).
23. Joung, S. J. & Hsu, H. H. Reproduction and embryonic development of the shortfin mako, *Isurus oxyrinchus* Rafinesque, 1810, in the Northwestern Pacific. *Zool. Stud.* **44,** 487–496 (2005).
24. Chang, J. H. & Liu, K. M. Stock assessment of the shortfin mako shark (*Isurus oxyrinchus*) in the Northwest Pacific Ocean using per recruit and virtual population analyses. *Fish. Res.* **98,** 92–101 (2009).
25. Semba, Y., Aoki, I. & Yokawa, K. Size at maturity and reproductive traits of shortfin mako, *Isurus oxyrinchus* , in the western and central North Pacific. *Mar. Freshw. Res.* **62,** 20–29 (2011).
26. Gilmore, R. G. Reproductive biology of lamnoid sharks. *Environ. Biol. Fishes* **38,** 95–114 (1993).
27. Mollet, H. F., Cliff, G., Pratt, H. L. & Stevens, J. D. Reproductive biology of the female shortfin mako, *Isurus oxyrinchus* Rafinesque, 1810, with comments on the embryonic development of lamnoids. *Fish. Bull.* **98,** 299–318 (2000).
28. Duffy, C. & Francis, M. P. Evidence of summer parturition in shortfin mako (*Isurus oxyrinchus*) sharks from New Zealand waters. *New Zeal. J. Mar. Freshw. Res.* **35,** 319–324 (2001).
29. Groeneveld, J. C. *et al.* Population structure and biology of shortfin mako, *Isurus oxyrinchus*, in the south-west Indian Ocean. *Mar. Freshw. Res.* **65,** 1045 (2014).

30. Natanson, L. J. *et al.* Validated age and growth estimates for the shortfin mako, *Isurus oxyrinchus*, in the North Atlantic Ocean. *Environ. Biol. Fishes* **77,** 367–383 (2006).
31. Bishop, S. D. H., Francis, M. P., Duffy, C. & Montgomery, J. C. Age, growth, maturity, longevity and natural mortality of the shortfin mako shark (*Isurus oxyrinchus*) in New Zealand waters. *Mar. Freshw. Res.* **57,** 143–154 (2006).
32. Barreto, R. R., Farias, W. K. T. de, Andrade, H., Santana, F. M. & Lessa, R. Age, Growth and Spatial Distribution of the Life Stages of the Shortfin Mako, *Isurus oxyrinchus* (Rafinesque, 1810) Caught in the Western and Central Atlantic. *PLoS One* **11,** e0153062 (2016).
33. Smith, S. E., Au, D. W. & Show, C. Intrinsic rebound potentials of 26 species of Pacific sharks. *Mar. Freshw. Res.* **49,** 663–678 (1998).
34. Campana, S. E., Marks, L. & Joyce, W. The biology and fishery of shortfin mako sharks (*Isurus oxyrinchus*) in Atlantic Canadian waters. *Fish. Res.* **73,** 341–352 (2005).
35. Ribot-Carballal, M. C., Galván-Magaña, F. & Quiñónez-Velázquez, C. Age and growth of the shortfin mako shark, Isurus oxyrinchus, from the western coast of Baja California Sur, Mexico. *Fish. Res.* **76,** 14–21 (2005).
36. Ardizzone, D. *et al.* Application of bomb radiocarbon chronologies to shortfin mako (*Isurus oxyrinchus*) age validation. *Environ. Biol. Fishes* **77,** 355–366 (2006).
37. Semba, Y., Nakano, H. & Aoki, I. Age and growth analysis of the shortfin mako, *Isurus oxyrinchus*, in the western and central North Pacific Ocean. *Environ. Biol. Fishes* **84,** 377–391 (2009).
38. Cerna, F. & Licandeo, R. Age and growth of the shortfin mako (*Isurus oxyrinchus*) in the south-eastern Pacific off Chile. *Mar. Freshw. Res.* **60,** 394–403 (2009).
39. Wells, D. R. J. *et al.* Age validation of juvenile Shortfin Mako (*Isurus oxyrinchus*) tagged and marked with oxytetracycline off southern California. *Fish. Bull.* **111,** 147–160 (2013).
40. Pratt Jr., H. L. & Casey, J. G. Age and Growth of the Shortfin Mako, *Isurus oxyrinchus* , Using Four Methods. *Can. J. Fish. Aquat. Sci.* **40,** 1944–1957 (1983).
41. Kai, M., Shiozaki, K., Ohshimo, S. & Yokawa, K. Growth and spatiotemporal distribution of juvenile shortfin mako (*Isurus oxyrinchus*) in the western and central North Pacific. *Mar. Freshw. Res.* **66,** 1176 (2015).
42. Doño, F., Montealegre-Quijano, S., Domingo, A. & Kinas, P. G. Bayesian age and growth analysis of the shortfin mako shark *Isurus oxyrinchus* in the Western South Atlantic Ocean using a flexible model. *Environ. Biol. Fishes* **98,** 517–533 (2015).
43. Kohler, N. E., Casey, J. G. & Turner, P. a. Length-weight relationships for 13 species of sharks from the western North Atlantic. *Fish. Bull.* **93,** 412–418 (1995).
44. Jensen, A. L. Beverton and Holt life history invariants result from optimal trade-off of reproduction and survival. *Can. J. Fish. Aquat. Sci.* **53,** 820–822 (1996).
